# Nexplanonectomy—the surgical removal of an embolized implanted contraceptive device: a case report and review of the literature

**DOI:** 10.1186/s13256-024-04547-7

**Published:** 2024-05-02

**Authors:** Edward K. Maybury, Zachary C. Affrin, Christian Popa, Max Fowler, Bryan D. Laliberte, Sarah C. Clarke

**Affiliations:** 1 Naval Medical Center San Diego, 34800 Bob Wilson Drive, San Diego, CA 92134 USA; 2 Department of Anesthesiology, Walter Reed National Military Medical Center, Bethesda, MD 20814 USA; 3 Darnall Medical Library, Walter Reed National Military Medical Center, Bethesda, MD 20814 USA

**Keywords:** Nexplanon, Implanted device migration, Segmentectomy, Single-lung ventilation, Video-assisted thoracoscopic surgery, Endovascular retrieval, Contraceptive removal

## Abstract

**Background:**

Nexplanon implants are a common hormonal contraceptive modality. Though rare, these devices can embolize into the injured wall of the basilic vein, through the right heart, and finally wedge itself into a pulmonary artery. With adherence to the arterial wall over time, it becomes less amenable to endovascular retrieval. Patients may present with symptoms mimicking a pulmonary embolism, or without any symptoms at all. In asymptomatic cases, endovascular retrieval and/or surgery is required when patients wish to begin having children prior to biological inactivity. The current literature showed as little as nine case reports detailing lung tissue removal in the aim of reversing a patient’s implanted contraceptive device.

**Case presentation:**

A 22-year-old asymptomatic active-duty Caucasian female presented for elective outpatient Nexplanon removal. The suspicion of possible implant migration arose when it was discovered to be non-palpable in her left arm. After plain film x-rays failed to localize the implant, a chest x-ray and follow-up Computed Tomography (CT) scan revealed that the Nexplanon had migrated to a distal branch of the left pulmonary artery. Due to the patient’s strong desires to begin having children, the decision was made for removal. Initial endovascular retrieval failed due to Nexplanon encapsulation within the arterial wall. Ultimately, the patient underwent a left video-assisted thoracoscopic surgery (VATS) for exploration and left lower lobe basilar S7–9 segmentectomy, which successfully removed the Nexplanon.

**Conclusions:**

Implanted contraceptive devices can rarely result in migration to the pulmonary vasculature. These radiopaque devices are detectable on imaging studies if patients and clinicians are unable to palpate them. An endovascular approach should be considered first to spare lung tissue and avoid chest-wall incisions, but can be complicated by encapsulation and adherence to adjacent tissue. A VATS procedure with single-lung ventilation via a double-lumen endotracheal tube allows surgeons to safely operate on an immobilized lung while anesthesiologists facilitate single-lung ventilation. This patient’s case details the uncommon phenomenon of Nexplanon migration, and the exceedingly rare treatment resolution of lung resection to remove an embolized device.

**Supplementary Information:**

The online version contains supplementary material available at 10.1186/s13256-024-04547-7.

## Introduction

Patients elect to receive and remove implantable contraceptive devices at many different stages of life, and for a myriad of reasons. Some are young without comorbidities, while others can be older with many health conditions to consider. Nexplanon is an estrogen-free hormonal implant measuring 4 cm in length and designed to be inserted into the upper arm for constant gradual administration of etonogestrel over 3 years. They are meant to be detected by direct palpation to the arm, where they are placed. Rarely, they can embolize through a large vein and travel through the right atrium, then the right ventricle, and end up in the pulmonary vasculature. As the vascular diameter narrows, the device can wedge into a pulmonary artery, preventing blood supply similar in nature to a pulmonary embolism. When these devices migrate, a patient can become suddenly symptomatic, gradually symptomatic, or not symptomatic as all [1, 5–8, 11, 14]. This varied presentation is a product of the ultimate end-location of the migrated device, and the patient’s overall health. Upon the discovery that a device has migrated, typically by failure to palpate the implant, imaging studies are employed, which reflects the actions taken in this case [1–15]. Following Computed Tomography (CT) chest imaging, the device was confirmed to be located in the left lower lobe, highly suspicious of migration via the left upper extremity (LUE) basilic vein. Herein, we present a rare case of Nexplanonectomy via partial lung lobectomy in a young, healthy female.

## Case description

**Fig. 1:**
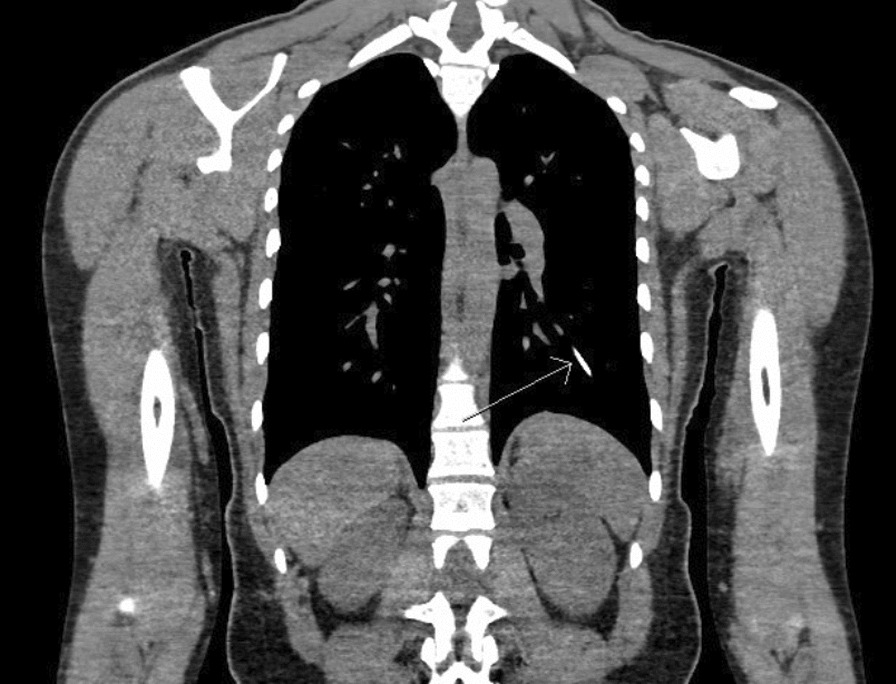
CT chest showing embolized Nexplanon (thin white arrow)

A 22-year-old healthy active-duty Caucasian female presented to her primary care clinic for removal of her implanted Nexplanon device approximately 1 year after placement at another facility. Palpation of her upper arm yielded no appreciable rod. Subsequent imaging of the upper arm and chest was concerning for the presence of a foreign body in the left lung field. CT imaging of the chest revealed the Nexplanon implant to be located in her left lower lobe (Fig. 1). At no time did she express respiratory symptoms, pain, or decrease in physical stamina either at rest or while exercising. There was concern for a loss of perfusion and destruction of the parenchyma, however, previously reported asymptomatic cases like this one have safely left devices in situ per the patient’s wishes. This option would alleviate the need for surgery, but would also prevent her from bearing children. Though she had been asymptomatic, she expressed a strong desire to start a family imminently, and did not wish to leave the implant in place and wait for it to become hormonally inactive. Thus, the decision for removal was made and the patient was scheduled for an endovascular procedure.

**Fig. 2:**
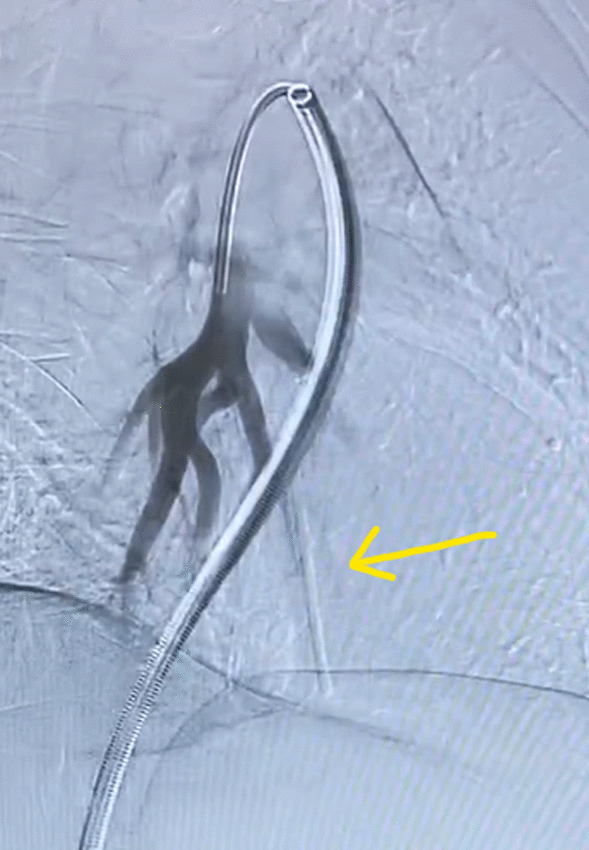
Interventional radiology image with contrast dye proximal to Nexplanon device (yellow arrow) series A

**Fig. 3:**
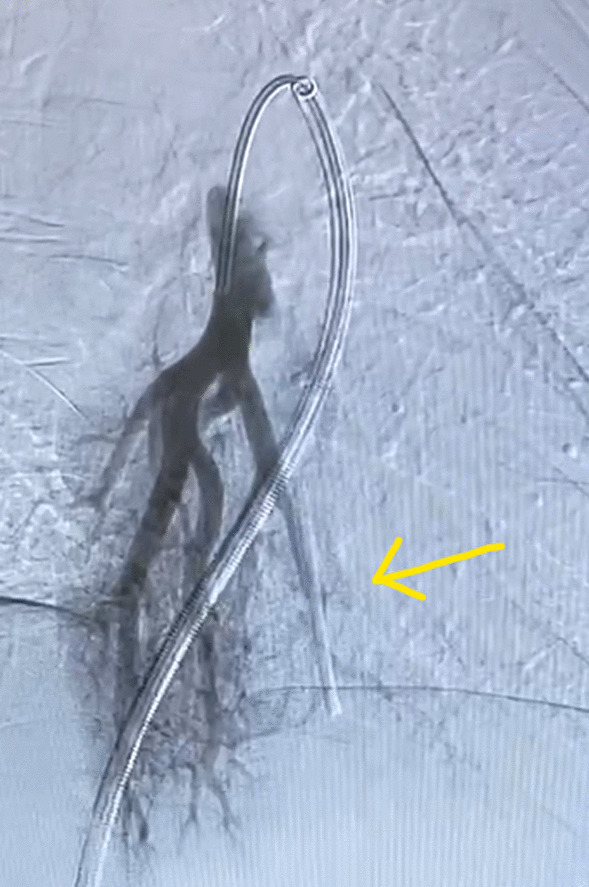
Interventional radiology image with contrast dye obstructed by Nexplanon device (yellow arrow) series A

**Fig. 4:**
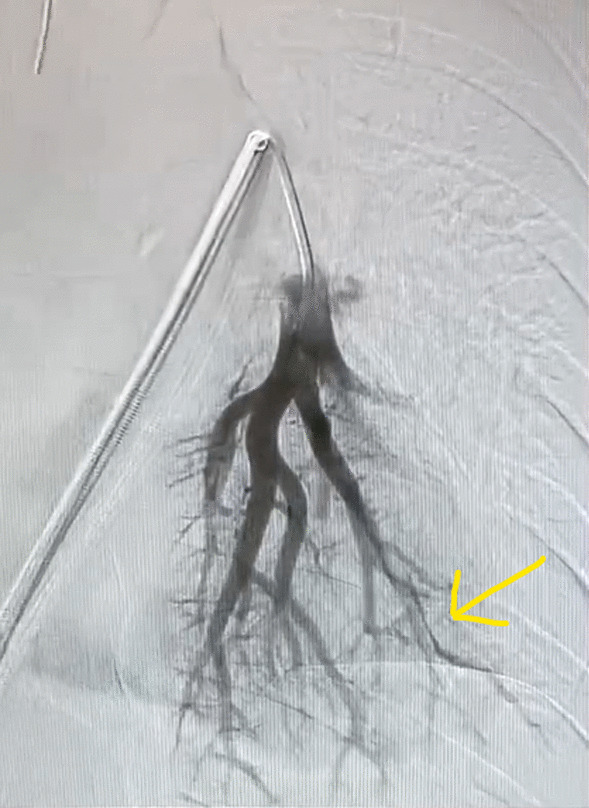
Interventional radiology image with contrast dye obstructed by Nexplanon device (yellow arrow) series B

**Fig. 5:**
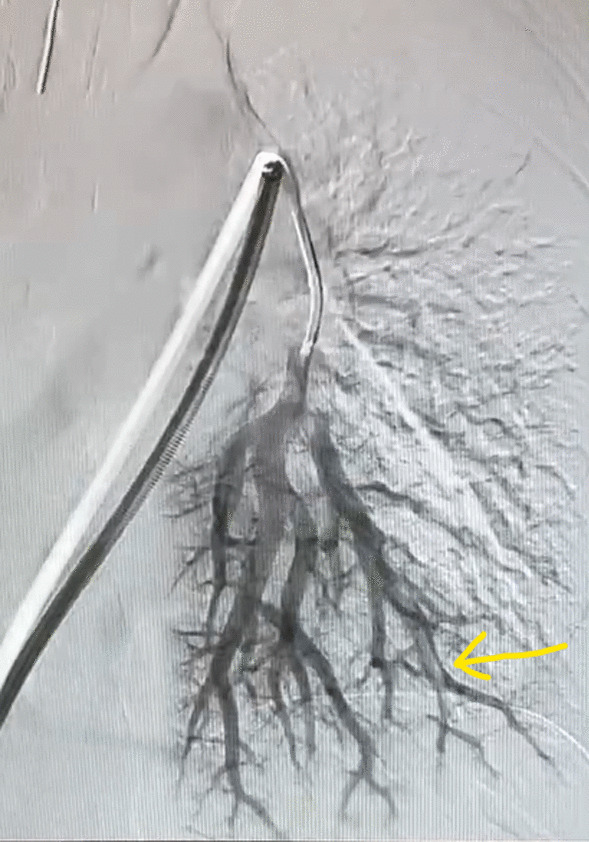
Interventional radiology image with contrast dye progressing through distal pulmonary arteries not obstructed by Nexplanon device (yellow arrow)

**Fig. 6:**
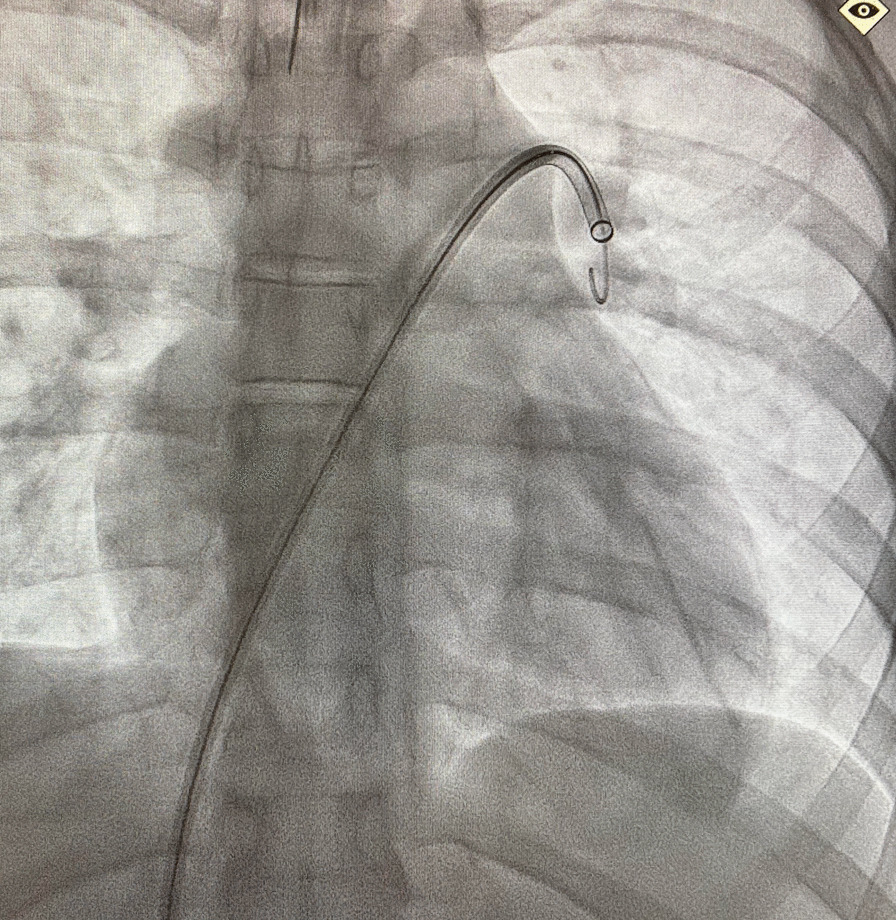
Interventional radiology image without contrast showing radiopaque Nexplanon device near the left heart border

**Fig. 7:**
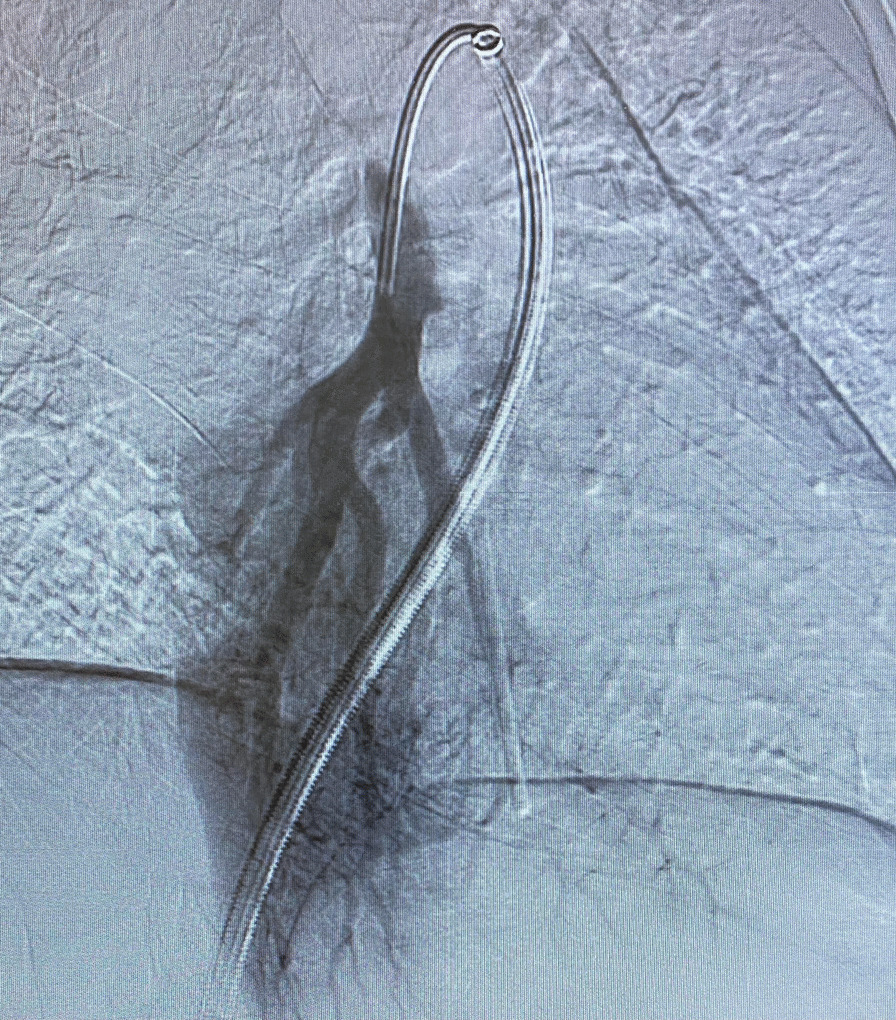
Interventional radiology image with contrast showing the Nexplanon device obstructing contrast flow to distal arterial vessels

The patient was taken to the interventional radiology suite, where she received a general anesthetic with endotracheal intubation. Interventional radiology attempted to remove the implant safely from the distal branch of the left pulmonary artery, via femoral vascular access. After numerous attempts there was a high suspicion that the Nexplanon had become encapsulated and was adhered to the wall of that artery, as the implant did not retract freely when snared (Figs. 2, 3, 4, 5, 6, 7, Additional file 1: Video S1, Additional file 2: Video S2, Additional file 3: Video S3). There was a concern for catastrophic bleeding should further attempts at endovascular removal be made. This new consideration led to the halting of this procedure and a discussion with the patient about how to proceed.

**Fig. 8:**
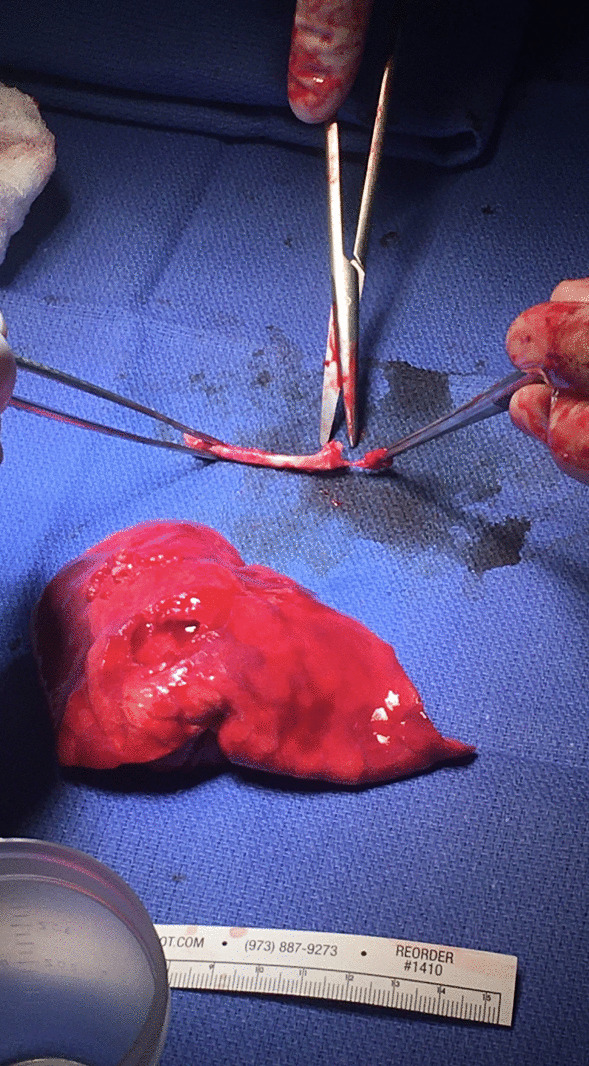
Segmentectomy with excised Nexplanon

Thoracic surgery was consulted and the patient underwent a left VATS for exploration and left lower lobe basilar S7–9 segmentectomy (Fig. 8). General anesthesia with a double lumen endotracheal tube was used to simultaneously allow for adequate respiration and isolated left lung deflation. The S6 superior segment of the left lower lobe was spared with preoperative and postoperative fiberoptic bronchoscopy. The procedure was uncomplicated, and the patient was transferred to the surgical intensive care unit for recovery. On post-operative day 1, she was ambulating and her pain was well controlled. She was transferred to a floor unit on post-operative day 2. On post-operative day 3, her chest tube was discontinued and she was discharged. At her 28-week and 45-week follow-up, she had continuing chronic left flank pain as well as chest pain with deep inspiration. She complied with her pain medication regimen and continued to follow up as needed.

## Discussion and conclusions

The estimated incidence of Nexplanon migration into pulmonary vasculature is 3.17 per 100,000 implants (95% CI 1.37 to 6.24) [12]. Statistics from the National Survey of Family Growth estimated the percentage of women aged 15–49 who have ever used contraceptive implants to be 5.6 from 2015 to 2017 (SE 0.50, *n* = 5594), and 5.8 from 2017 to 2019 (SE 0.46, *n* = 6141) [9]. The above case is highly unusual, both with respect to the device migrating from brachial subcutaneous tissue to the vasculature of the lung, and the ultimate action needed for removal. Of the 55 reported cases of contraceptive device migration from the upper arm into the pulmonary vasculature, much can be gathered. The first consideration would be to attempt an endovascular retrieval, lessening the need for an open procedure. 21 of the 55 cases detail successful retrieval of the pulmonary embolized Nexplanon, without major complications. Of the 7 unsuccessful endovascular procedures, 5 moved forward with successful VATS, without major complications. The remaining 2 were left in situ. Of the total cases, 11 elected for successful VATS without an endovascular attempt. 13 cases did not move forward with any retrieval attempts, and left their devices in situ. 3 cases have unknown surgical considerations or outcomes. These findings can be seen in Tables 1, 2, 3.

**Table 1:** Synopsis of case reports concerning pulmonary embolized Nexplanons and retrieval method

Outcome	(*n*)	% total (%)
Successful endovascular retrieval	21	38
No operation (left in situ)	13	23
Straight to VATS without endovascular attempt	11	20
Unsuccessful endovascular retrieval; successful VATS	6^a^	11
Unsuccessful endovascular retrieval (left in situ)	2	4
Unknown	3	5
Total	56	

^a^Includes our case

**Table 2:** Reported cases of implanted contraceptive devices migrating into the pulmonary arteries (2014–2017)

Pub. year	Authors	Title	Age	Migration site	Operative results	Case notes
2014	Patel, A.	Contraceptive implant embolism into the pulmonary artery^a^	36	LLL	Unwanted side effects improved. Pt declined further intervention	–
2015	D'Journo, X. B.	Intravascular pulmonary migration of a subdermal contraceptive implant [8]	20	LLL	Implant too distal for EI. SRWC via L basal trisegmentectomy w/thoracoscopy	Regular menorrhagia s/p implantation, decided to remove it 15 days later
2015	Heudes, P-M	Migration of a contraceptive subcutaneous device into the pulmonary artery. Report of a case [7]	18	Apical RUL	SRWC via EI	Immediate local hematoma s/p implantation. Elected for removal 5 months later 2/2 pain. CXR and subsequent CT chest revealed the migration
2015	O'Brien, A.	Subdermal contraceptive implant embolism to a pulmonary artery [6]	23	LLL	EI unsuccessful (too endothelized/fibrosed after 2 years). Pt declined further intervention	Pt presented to the ED w/dyspnea and PTX on CXR. Unless recognized quickly, successful endovascular removal is unlikely
2017	Barlow-Evans, R.	Migration of a Nexplanon contraceptive implant to the pulmonary artery [3]	17	LLL	EI not attempted (too endothelized/fibrosed after 2 years). Mutual agreement to leave in situ	Alternative contraception advised at the end of its 3 year licensed duration
2017	Choi, J. H.	Migration of a contraceptive subdermal device into the lung [11]	37	Posterior basal LLL	SRWC via MT	Pt experienced irregular vaginal bleeding for 2 years prior to removal
2017	Chung, M.	Endovascular retrieval of Nexplanon from the distal pulmonary artery^a^	23	LLL	SRWC via EI	–
2017	Gallon, A.	Looking for a lost subdermal contraceptive implant? Think about the pulmonary artery^a^	29	RLL	SRWC via EI	–
2017	Kew, E. P.	Migration of contraceptive implant into the left pulmonary arterial system [14]	18	LLL	Pt declined intervention	Long-term effects of leaving an implant in the pulmonary arterial system are unknown
2017	Thomas, P. A.	Contraceptive implant embolism into the pulmonary artery: thoracoscopic retrieval [10]	18	LLL	SR via thoracoscopic arteriotomy. Post-op complicated by pleural clotting. VATS revision. No active bleeding detected at arteriotomy site. No complaints at 1 year f/u	–

*EI* endovascular intervention, *SRWC* successful retrieval without complications, *Pt* patient, *SR* successful retrieval, *VATS* video-assisted thoracoscopic surgery, *PA* pulmonary artery, *LPA* left-sided pulmonary artery, *RPA* right-sided pulmonary artery, *f/u* follow up, *LLL* left lower lobe, *RLL* right lower lobe, *LUL* left upper lobe, *RUL* right upper lobe, *RML* right middle lobe, *s/p* status post, *2/2* secondary to, *MT* mini-thoracotomy

^a^Additional references available upon request

**Table 3:** Reported cases of implanted contraceptive devices migrating into the pulmonary arteries (2018–2022)

Pub. year	Authors	Title	Age	Migration site	Operative results	Case notes
2018	Gao, G. T.	Embolization of a contraceptive implant into the pulmonary vasculature in an adolescent female [15]	16	LLL	SRWC via EI	Pt presented to peds ER with subjective dyspnea
2018	Wilcox, K. K.	Endovascular retrieval of contraceptive implant embolized to pulmonary artery^a^	22	RLL	SRWC via EI	–
2018	Akhtar, M. M.	Percutaneous extraction of an embolized progesterone contraceptive implant from the pulmonary artery^a^	31	RLL	SRWC via EI	Pt sought elective removal 1 year after implantation without complaints
2019	Carlos-Alves, M.	Lung migration of contraceptive Implanon NXT^a^	31	LLL	SRWC via VATS	–
2019	Cerato, A.	Migration of contraceptive implant into the pulmonary artery [13]	16	Latero-basal LLL	EI not attempted (complete vascular retraction around the device). Pt declined further intervention	–
2020	Simon, C.	Incidence and characteristics of intravascular pulmonary migration of etonogestrel implants: a French nationwide study [12]	various (27)	LPA (9), RPA (4), unspecified (14)	SRWC via EI (10), SRWC via surgery (5), left in situ (9), unspecified (3)	–
2020	Hindy, J. R.	Nexplanon migration into a subsegmental branch of the pulmonary artery: a case report and review of the literature [4]	26	lateral RML	SRWC via EI	No clear guidelines have been published for preventing this challenging complication
2021	Carraro do Nascimento, V.	Aspiration technique for percutaneous endovascular retrieval of contraceptive device embolized to the pulmonary vasculature^a^	46	LLL	SRWC via EI	–
2021	Wali, A.	Contraceptive implant migration to the lung^a^	27	LLL	MT and arteriotomy unsuccessful. SRWC via segmentectomy	Significant arterial wall endothelialization
2022	Tricard, J.	Transparenchymal thoracoscopic retrieval of a contraceptive implant pulmonary embolism [5]	21	RLL	SRWC via thoracoscopic arteriotomy	Removal occurred 4 years after implantation
2022	Kafi Mallak, F.	Migration of a subdermal contraceptive implant into a subsegmental pulmonary artery and etonogestrel serum concentration over time—a case report [2]	24	Posterior basal LLL	EI unsuccessful. Implant remains in situ	Etonogestrel and estradiol concentrations were followed over time
2022	Clermidy, H.	Management of etonogestrel implant migration into the pulmonary artery [1]	Various (8)	4 LLL, 1 RLL, 1 RUL, 1 LUL, 1 RML	SRWC via EI (3). EI unsuccessful, SRWC via MT (5)	5 pts had variations of CP and dyspnea. Angiography displayed arterial thrombosis distal to the implant

*EI* endovascular intervention, *SRWC* successful retrieval without complications, *Pt* patient, *SR* successful retrieval, *VATS* video-assisted thoracoscopic surgery, *PA* pulmonary artery, *LPA* left-sided pulmonary artery, *RPA* right-sided pulmonary artery, *f/u* follow up, *LLL* left lower lobe, *RLL* right lower lobe, *LUL* left upper lobe, *RUL* right upper lobe, *RML* right middle lobe, *s/p* status post, *2/2* secondary to, *MT* mini-thoracotomy

^a^Additional references available upon request

The impetus for suspicion of migration of a Nexplanon is varied. Some patients may be symptomatic, and exhibit presentation signs of pulmonary embolism in the event of an implant migration. Our patient was quite athletic, and as the Nexplanon had embolized to a distal branch of the left pulmonary artery, the loss of perfusion to that segment of the lung did not result in symptoms.

Following placement, patients are instructed on how to palpate for their Nexplanon device. They and their clinicians should be able to reliably relocate it at any time. Patients are also instructed to seek evaluation should they notice any irregularities with routine palpations. Despite the absence of guidelines pertaining to the work-up of a possibly embolized Nexplanon [4], these devices are radio-opaque and plain films of the arm and then the chest should be considered upon failure to palpate the device. Findings on a chest x-ray of possible pulmonary migration should be followed up by CT imaging to further characterize the location. A minimally invasive approach, such as endovascular retrieval should then be considered if the patient strongly desires implant removal, or if the patient is symptomatic because of the embolized device. Endovascular approaches have the benefit of sparing lung tissue and avoiding the pain of chest-wall incisions, but can be complicated by the Nexplanon’s tendency to encapsulate and adhere to adjacent tissue [1–3, 6, 12, 13]. This encapsulation is by design and is meant to prevent a Nexplanon from leaving its primary placement site, but can be problematic if it occurs after the device has moved. As seen in this case, this encapsulation can occur at the wall of a vessel, and thus make the device adherent and not amenable to endovascular removal. There are only a handful of studies correlating the duration of time since implantation in relation to successful endovascular retrieval [1, 3, 6, 12]. Utilizing patient history in regard to how long the implant has been in place is not a reliable measure to predict endovascular fibrosis and trapping. Our patient did not seek consultation until a desire for removal approximately 1 year following implantation. In asymptomatic patients, like ours, it is unclear whether the embolization occurred closer to the time of implantation or some time thereafter. In 38% of the reported cases, endovascular retrieval proved successful. In rare cases, such as this one, a VATS operation will be necessary after a failed endovascular procedure (11%). It is key that patients are aware of the potential need for two procedures when making the decision to remove an embolized Nexplanon.

### Supplementary Information

**Additional file 1: Video S1.** Nexplanon obstructing contrast flow (right side of screen).

**Additional file 2: Video S2.** Nexplanon obstructing contrast flow (alternate angle).

**Additional file 3: Video S3.** Nexplanon visible while contrast applied to a nearby uninvolved pulmonary artery.

## Data Availability

The datasets used and/or analyzed during this case report and literature review are publicly available, as well as available from the corresponding author on reasonable request.

## Abbreviations

VATS: Video-assisted thoracoscopic surgery
CT: Computed tomography
LUE: Left upper extremity
